# Two Novel Bacteriophages Improve Survival in *Galleria mellonella* Infection and Mouse Acute Pneumonia Models Infected with Extensively Drug-Resistant *Pseudomonas aeruginosa*

**DOI:** 10.1128/AEM.02900-18

**Published:** 2019-04-18

**Authors:** Jongsoo Jeon, Dongeun Yong

**Affiliations:** a Department of Laboratory Medicine and Research Institute of Bacterial Resistance, Yonsei University College of Medicine, Seoul, Republic of Korea; Centers for Disease Control and Prevention

**Keywords:** *Galleria mellonella* infection, *Pseudomonas aeruginosa*, *Siphoviridae*, bacteriophage, biofilm, mouse acute pneumonia, phage therapy

## Abstract

Extensively drug-resistant Pseudomonas aeruginosa (XDR-PA) is a life-threatening pathogen that causes serious global problems. Here, we investigated two novel P. aeruginosa bacteriophages (phages), Bϕ-R656 and Bϕ-R1836, *in vitro*, *in silico*, and *in vivo* to evaluate the potential of phage therapy to control XDR-PA clinical strains. Bϕ-R656 and Bϕ-R1836 belong to the *Siphoviridae* family and exhibited broad host ranges which could lyse 18 (64%) and 14 (50%) of the 28 XDR-PA strains. In addition, the two phages showed strong bacteriolytic activity against XDR-PA host strains from pneumonia patients. The whole genomes of Bϕ-R656 and Bϕ-R1836 have linear double-stranded DNA of 60,919 and 37,714 bp, respectively. The complete sequence of Bϕ-R656 had very low similarity to the previously discovered P. aeruginosa phages in GenBank, but phage Bϕ-R1836 exhibited 98% and 91% nucleotide similarity to *Pseudomonas* phages YMC12/01/R24 and PA1/KOR/2010, respectively. In the two *in vivo* infection models, treatment with Bϕ-R656 and Bϕ-R1836 enhanced the survival of Galleria mellonella larvae (50% and 60%, respectively) at 72 h postinfection and pneumonia-model mice (66% and 83%, respectively) at 12 days postinfection compared with untreated controls. Treatment with Bϕ-R656 or Bϕ-R1836 also significantly decreased the bacterial load in the lungs of the mouse pneumonia model (>6 log_10_ CFU and >4 log_10_ CFU, respectively) on day 5.

**IMPORTANCE** In this study, two novel P. aeruginosa phages, Bϕ-R656 and Bϕ-R1836, were evaluated *in vitro*, *in silico*, and *in vivo* for therapeutic efficacy and safety as an alternative antibacterial agent to control XDR-PA strains collected from pneumonia patients. Both phages exhibited potent bacteriolytic activity and greatly improved survival in G. mellonella larva infection and a mouse acute pneumonia model. Based on these results, we strongly predict that these two new phages could be used as fast-acting and safe alternative biological weapons against XDR-PA infections.

## Abstract (precis)

In this study, two novel P. aeruginosa phages, Bϕ-R656 and Bϕ-R1836, were evaluated *in vitro*, *in silico*, and *in vivo* for therapeutic efficacy and safety as an alternative antibacterial agent to control XDR-PA strains collected from pneumonia patients. Both phages exhibited potent bacteriolytic activity and greatly improved survival in G. mellonella larva infection and a mouse acute pneumonia model. Based on these results, we strongly predict that these two new phages could be used as fast-acting and safe alternative biological weapons against XDR-PA infections.

## INTRODUCTION

The increase in multidrug-resistant (MDR) pathogens, in particular the ESKAPE (Enterococcus faecium, Staphylococcus aureus, Klebsiella pneumoniae, Acinetobacter baumannii, Pseudomonas aeruginosa, and *Enterobacter* species) pathogens, is a major public health concern around the globe (1). Pseudomonas aeruginosa is an opportunistic pathogen that has emerged as a major threat, causing a variety of acute infections in the hospital environment (2). This bacterium is associated with ventilator-associated pneumonia, burn wound infections, osteochondritis, and urinary tract infections. In particular, extensively drug-resistant P. aeruginosa (XDR-PA) causes chronic lung infections in immunocompromised patients with cystic fibrosis (CF), and has contributed to high morbidity and mortality in these patients (3, 4). XDR was defined as nonsusceptibility to at least one agent in all but two or fewer antimicrobial categories (i.e., strains remain susceptible to only one or two categories) (5).

These organisms are notoriously difficult to treat and have led to treatment failures because of their intrinsic (e.g., efflux pumps, β-lactamases, and carbapenemases) and acquired (e.g., horizontal transfer and mutational resistance) resistance mechanisms to a wide variety of antibiotics (6, 7). Therefore, novel therapeutic approaches for treating multidrug-resistant pathogen infections, including P. aeruginosa, are urgently needed. With the rapid emergence of antibiotic-resistant strains of pathogenic bacteria worldwide, the use of bacteriophages (phages), which are viruses that infect bacteria, has been renewed as an alternative therapeutic option.

The application of phages as therapeutic agents to control pathogens is not a new concept. Since the discovery of phages by Frederick Twort in 1915 and Félix d’Herelle in 1917, phage therapy was actively performed in the former Soviet Union and in Eastern Europe for decades, and it is still used today as a promising alternative to treat several infections, especially in Georgia (8–10). Phages have several advantages over conventional antibiotics, such as their easy isolation, cost-effectiveness, and absolute host specificity, as well as the fact that they are obligate bacterial viruses that self-multiply, do not damage the normal microbiota, and have no known adverse side effects (11–14).

Various animal studies have been conducted to evaluate the safety and efficacy of phage therapy for serious clinical pathogens, including Acinetobacter baumannii (15), Pseudomonas aeruginosa (16, 17), Staphylococcus aureus (18), Enterococcus faecium (19), Escherichia coli (20), and Klebsiella pneumoniae (21). In 2006, phage cocktails targeting Listeria monocytogenes were first approved by the U.S. Food and Drug Administration as antibacterial food additives to be used on ready-to-eat meat and poultry products (22). Recently, the Phagoburn project (http://www.phagoburn.eu/) of clinical trials conducted by various European countries and pharmaceutical companies aimed to develop phage cocktails to treat infectious diseases caused by E. coli and P. aeruginosa in burn patients. The evolution of phage-resistant bacteria is one of the major hurdles associated with phage therapies (23). Accordingly, therapeutic use of phage cocktails has been recently proposed to solve the limitation in phage host ranges and reduce the development of phage-resistant mutants (23). However, isolation and identification of novel, well-characterized single phages with a broader antibacterial spectrum and strong lytic activity are vitally important for generation of phage cocktails (24).

In recent studies, the therapeutic potential of P. aeruginosa phages against multidrug-resistant P. aeruginosa was investigated in applications using phages in either single or cocktail treatments (14, 25, 26), synergy of phage therapy with antibiotics (27), and disruption of pseudomonal biofilms by phages in both *in vitro* and *in vivo* models (28). Especially, in previous studies for human trials of P. aeruginosa phages as therapeutic agents, Wright et al. applied phage therapy to treat P. aeruginosa-associated chronic otitis (29) and Chan et al. reported therapeutic application of phage on an aortic Dacron graft with chronic infection caused by P. aeruginosa (30).

To date, the complete genomes of approximately 76 *Siphoviridae* phages infecting P. aeruginosa are available in the National Center for Biotechnology Information database (https://www.ncbi.nlm.nih.gov/genome/; 1 October 2018). Several studies concerning the therapeutic potential of phages against P. aeruginosa have been reported, including some studies using phage therapy for pulmonary infections caused by multidrug-resistant (MDR) P. aeruginosa in animal models (26, 31, 32). However, there have been few studies on the activity of P. aeruginosa phages on XDR-PA isolates from patients with pneumonia. In this work, we isolated and characterized two novel P. aeruginosa phages, Bϕ-R656 and Bϕ-R1836, and evaluated their therapeutic potential to control XDR-PA in patients with pneumonia using Galleria mellonella infection and a mouse acute pneumonia model.

## RESULTS

### *In vitro* characterization of the two phages.

**FIG 1:**
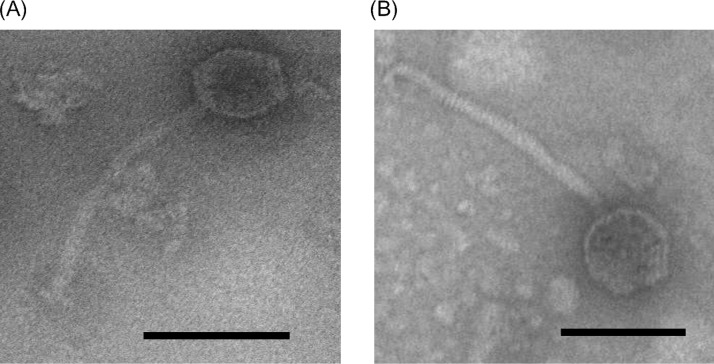
Morphological images of Bϕ-R656 (A) and Bϕ-R1836 (B) acquired using transmission electron microscopy. Bar = 100 nm.

**FIG 2:**
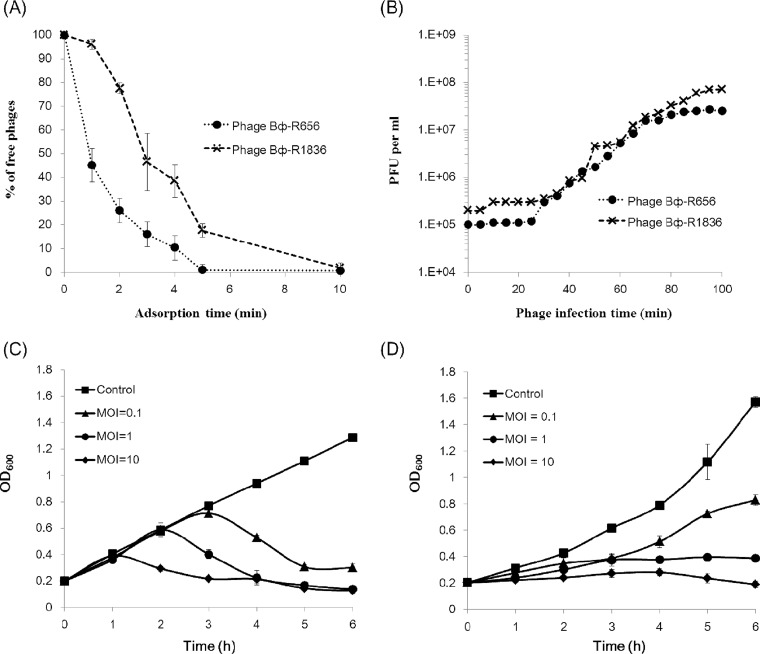
*In vitro* characterization of Bϕ-R656 and Bϕ-R1836. (A and B) Adsorption rates (A) and one-step growth curves (B) of Bϕ-R656 (●) and Bϕ-R1836 (×). (C and D) Kinetics of cell lysis by Bϕ-R656 (C) and Bϕ-R1836 (D) in XDR P. aeruginosa strains YMC11/02/R656 and YMC11/11/R1836, respectively. The host bacteria were infected with Bϕ-R656 or Bϕ-R1836 at MOIs of 0.1, 1, and 10. The data represent the mean ± standard deviation from triplicate experiments.

The morphological characteristics determined using TEM indicated that the two P. aeruginosa phages Bϕ-R656 and Bϕ-R1836, isolated from hospital sewage, belong to the *Siphoviridae* family, and both phages had an icosahedral head approximately 62 nm in diameter and a tail approximately 195 nm in length (*n* = 10) (Fig. 1). The host spectra of phages Bϕ-R656 and Bϕ-R1836 were determined using spot testing and efficiency of plating (EOP) assays, and the phages formed clear or turbid plaques of 2 to 3 mm against 18 (64%) and 14 (50%) of the 28 XDR-PA clinical strains tested, respectively (see Table S1 in the supplemental material). Nineteen of 28 XDR-PA clinical isolates formed plaques when exposed to Bϕ-R656 and Bϕ-R1836. The *in vitro* sensitivities of phages were not equal in 16 of 28 strains. Nine out of 28 strains were insensitive to both phages. Phages Bϕ-R656 and Bϕ-R1836 had adsorption rates of 99% within 5 min and 10 min, respectively (Fig. 2A) and a burst size of 253 and 499 PFU per infected cell, and both phages had a latent period of 30 min (Fig. 2B).

In cell lysis and stability analyses, phages Bϕ-R656 (Fig. 2C) and Bϕ-R1836 (Fig. 2D) inhibited bacterial growth at all MOIs at 6 h, except for Bϕ-R1836 at an MOI of 0.1. In particular, both phages exhibited strong lysis activity at an MOI of 10 (OD_600_ = 0.16 and OD_600_ = 0.18, respectively, at 6 h). The phages maintained a high stability of >99% at 4°C for 1 h and exhibited stabilities of 34% and 64%, respectively, at 50°C for 1 h (Fig. S2). In the pH stability tests, the phages had stabilities of 53% and 84%, respectively, on day 30 at pH 7. Moreover, phage Bϕ-R1836 retained a notable stability of 52% at pH 4 and 75% at pH 10 on day 30, but phage Bϕ-R656 showed relatively low stabilities of 33% at pH 4 and 12% at pH 10 on that day (Fig. S3).

In biofilm-disruption ability, Bϕ-R656 (Fig. S4A and B) and Bϕ-R1836 (Fig. S4C and D) also significantly reduced the host bacterial biofilms from 0.98 to 0.10 and from 1.21 to 0.26 at OD_590_ (****, *P* < 0.0001).

### *In silico* analysis of the two phages.

**FIG 3:**
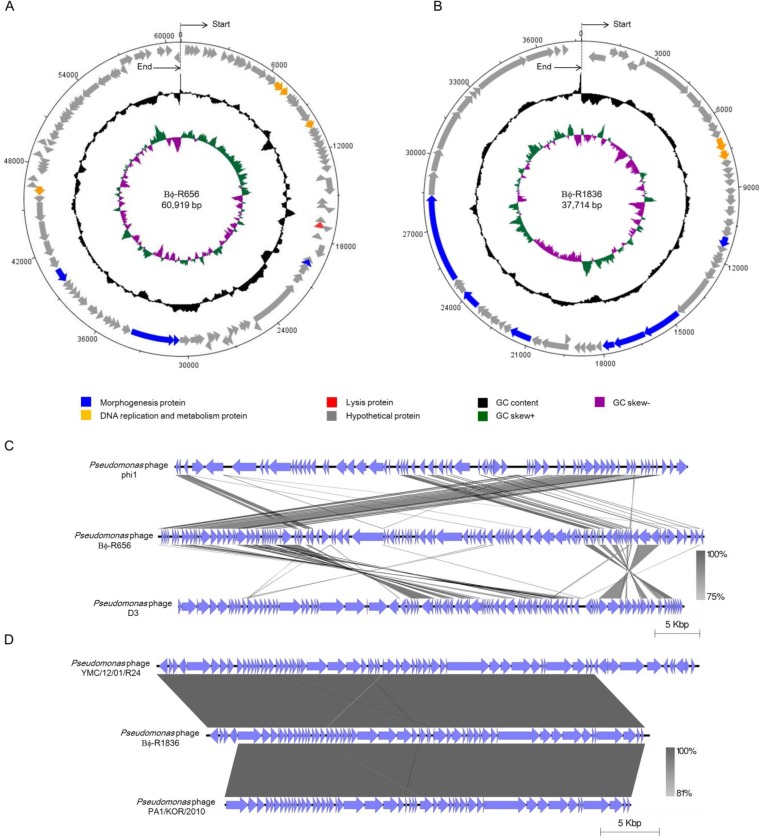
Genome organization of Bϕ-R656 (A) and Bϕ-R1836 (B) and genome comparisons of Bϕ-R656 (C) and Bϕ-R1836 (D) with their closest related bacteriophages. Circular maps (A and B) prepared using DNAPlotter. The outer ring shows the ORFs of the phages and their transcription direction. The inner rings indicate the GC content (black) and GC skew (+, green; −, purple). The genome alignments (C and D) of the bacteriophages were prepared using Easyfig software, version 2.1.

The whole genomes of phages Bϕ-R656 and Bϕ-R1836 were sequenced using a 454 GS Junior sequencer. The organization of the genomes is represented in Fig. 3A and B in a circular form using DNAPlotter, and their putative open reading frames (ORFs) are listed in Tables S2 and S3. The complete genome of each phage has a linear double-stranded DNA, with Bϕ-R656 having 60,919 bp (31,270 read lengths and 238-fold coverage) and a G+C content of 58.7% and Bϕ-R1836 having 37,714 bp (2,498 read lengths and 267-fold coverage) and a G+C content of 64.25%. Bioinformatics analysis of both phage genomes identified 113 and 59 putative ORFs, respectively, and only 9 ORFs exhibited homology with genes of other phages with annotated functions in GenBank. The annotated ORFs of both phages contain morphogenesis genes (phage Bϕ-R656: *orf* 45, *orf* 63, *orf* 64, and *orf* 76; Bϕ-R1836: *orf* 23, *orf* 30, *orf* 31, *orf* 32, *orf* 40, *orf* 46, and *orf* 49), phage DNA replication and metabolism genes (phage Bϕ-R656: *orf* 17, *orf* 18, *orf* 23, and *orf* 80; Bϕ-R1836: *orf* 12 and *orf* 13), and lysis genes (phage Bϕ-R656: *orf* 40). Also, phage Bϕ-R656 had one predicted tRNA (tRNA-Gly), while phage Bϕ-R1836 had no identified tRNAs. The complete genomes of phages Bϕ-R656 and Bϕ-R1836 were compared to other P. aeruginosa phage genomes using BLASTN from the National Center for Biotechnology Information (NCBI). The complete sequence of phage Bϕ-R656 had <16% sequence similarity (BLAST E value cutoff of 0.1) with those of P. aeruginosa phage phi1 (GenBank accession number KT887557.1) and phage D3 (GenBank accession number AF165214.2) but was not significantly similar to that of other previously reported P. aeruginosa phages (Fig. 3C). The genome sequence of phage Bϕ-R1836 had 98% and 91% nucleotide similarity (BLAST E value cutoff of 0.1) to P. aeruginosa phages YMC12/01/R24 (GenBank accession number MH643778.1) and PA1/KOR/2010 (GenBank accession number HM624080.1), respectively (Fig. 3D). Although most of the phage ORFs were putative genes, genes related to toxin and lysogeny-related genes in the two phage genomes were not detected by the NCBI database.

### *In vivo* efficacy of the two phages in Galleria mellonella model.

**FIG 4:**
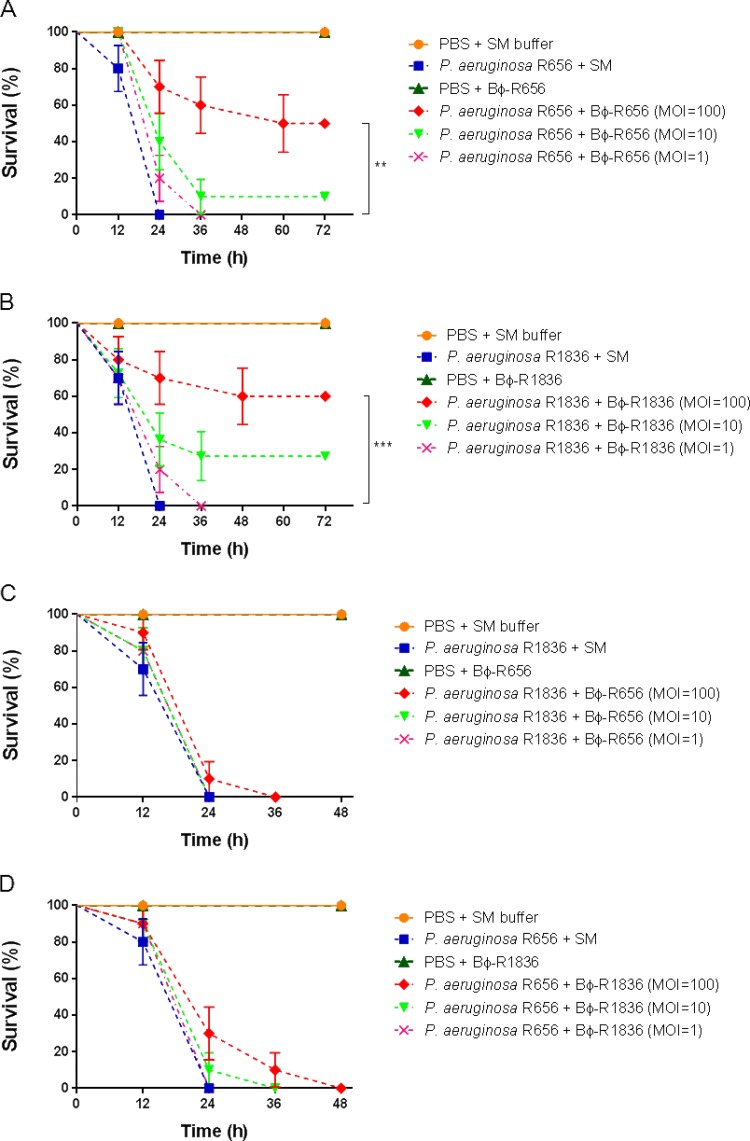
*In vivo* efficacy of Bϕ-R656 and Bϕ-R1836 against XDR P. aeruginosa YMC11/02/R656 and YMC11/11/R1836 strains in a Galleria mellonella infection model. Survival curves of G. mellonella larvae treated with Bϕ-R656 (A and C) or Bϕ-R1836 (B and D) at an MOI of 100 (1 × 10^7^ PFU/ml), 10 (1 × 10^6^ PFU/ml), or 1 (1 × 10^5^ PFU/ml) 1 h postinfection (1 × 10^5^ CFU/ml). G. mellonella larvae were monitored at 12-h intervals for 48 or 72 h. The percentage of G. mellonella survival was determined using the log rank (Mantel Cox) test (**, *P* = 0.0022; ***, *P* = 0.0003). The data represent the mean ± standard deviations (error bars) from three independent experiments with 10 animals per treatment.

Galleria mellonella larvae were used as a surrogate animal infection model to evaluate the *in vivo* therapeutic efficacy of two P. aeruginosa phages, Bϕ-R656 and Bϕ-R1836, against XDR-PA clinical strains. As shown in Fig. 4A and B, the G. mellonella larvae infected with strains YMC11/02/R656 (1 × 10^5^ CFU/ml) and YMC11/11/R1836 (1 × 10^5^ CFU/ml) showed 100% mortality at 24 h, and treatment with phages Bϕ-R656 (Fig. 4A) and Bϕ-R1836 (Fig. 4B) (1 × 10^7^ PFU/ml, MOI = 100, 1 h postinfection) resulted in statistically significantly improvement of the survival rate to 50% (**, *P* = 0.0022) and 60% (***, *P* = 0.0003), respectively, at 72 h against each host XDR-PA strain. The survival rates of the phage-treated groups at an MOI of 10 were 10% and 27%, respectively, at 72 h but showed no significant differences from the bacterium-infected groups, and the phage-treated groups at an MOI of 1 at 1 h postinfection showed 100% mortality at 36 h. Treatment with phages Bϕ-R656 (Fig. 4C) and Bϕ-R1836 (Fig. 4D) at an MOI of 100 against each other’s host P. aeruginosa strain showed survival rates of only 10% and 30%, respectively, at 24 h. The larvae injected with buffer (PBS + SM) or phage only (1 × 10^7^ PFU) showed no mortality up to 72 h.

The histology of larval tissues was determined by staining at 72 h of *in vivo* efficacy assays and was analyzed to determine the therapeutic effects of phages Bϕ-R656 (Fig. S5A) and Bϕ-R1836 (Fig. S5B). Infected larval tissues (1 × 10^5^ CFU/ml) showed melanized nodules in many areas, including the fat body well and the muscle layer, but tissues of phage-treated larvae (MOI = 100, 1 h postinfection) did not demonstrate notable melanization or damage. The tissues of larvae injected with phage only (1 × 10^7^ PFU/ml) or buffer (PBS + SM) also showed no toxicity or damage (Fig. S5).

### Therapeutic efficacy of the two phages in a mouse model of acute pneumonia.

**FIG 5:**
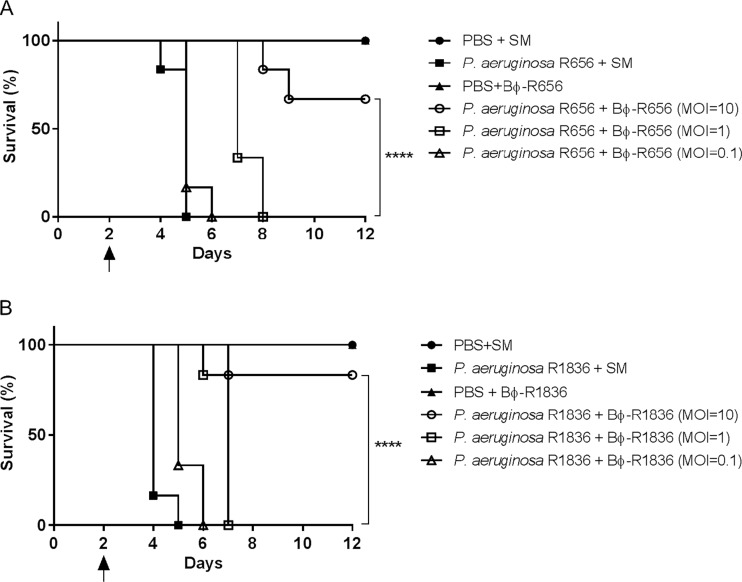
Therapeutic efficacy of Bϕ-R656 (A) and Bϕ-R1836 (B) in a mouse model of acute pneumonia caused by XDR P. aeruginosa YMC11/02/R656 and YMC11/11/R1836 strains. Female C57BL/6 mice were divided into the following six groups (*n* = 6 per group): buffer-only inoculation (PBS + SM) group, bacterium-only infection (1 × 10^8^ CFU/ml) group, phage-only treatment (1 × 10^9^ PFU/ml) group, and phage treatment (MOI = 10, 1, and 0.1) groups 4 h postinfection. The black arrow indicates a second cyclophosphamide (CP) administration and the first bacterial infection. Log rank (Mantel-Cox) test (****, *P* < 0.0001).

To evaluate the *in vivo* therapeutic potential and safety of the phages, we used a mouse model of acute pneumonia caused by XDR-PA host strains. Phages Bϕ-R656 (Fig. 5A) and Bϕ-R1836 (Fig. 5B) significantly improved the survival rate of mice with acute pneumonia. The phage-treated groups (MOI = 10 at 4 h postinfection) showed high survival rates of 66% and 83%, respectively, on day 12, but animals in the bacterium-infected groups all died by day 3 postinfection. The groups injected with phage only (1 × 10^7^ PFU/ml) or buffer (PBS + SM) exhibited no mortality or loss of body weight (Fig. S6).

### Histological changes and cytokine levels.

**FIG 6:**
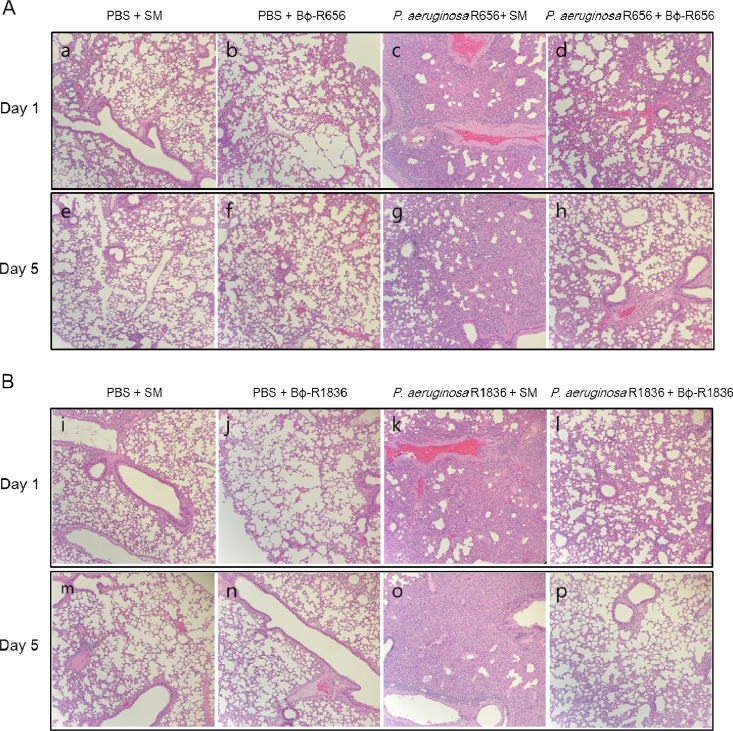
Histological analysis of lung sections from mice treated with Bϕ-R656 (A) or Bϕ-R1836 (B). Mice were sacrificed on either day 1 or 5. Lung sections were stained with hematoxylin and eosin and observed using an optical microscope at a magnification of ×10.

The histological changes and immune responses due to phage therapy were assessed using H&E staining and cytokine (TNF-α and IL-6) analysis, respectively. The bacterium-infected group had more severe hemorrhaging in the alveolar space and alveolar wall thickening on days 1 and 5 than the phage-treated groups. The phage-only-injected group showed histological changes on days 1 and 5 similar to those of the buffer (PBS + SM)-injected group (Fig. 6).

The levels of TNF-α and IL-6 in the lungs of bacterium-infected or phage-treated mice were comparatively higher than in the buffer-injected group on day 1 (****, *P* < 0.0001; **, *P* = 0.0024). However, these cytokine levels were significantly reduced at day 5, and no significant differences were detected among the experimental groups at that time point (Fig. S7 and S8).

### Bacterial counts in mouse lungs.

**FIG 7:**
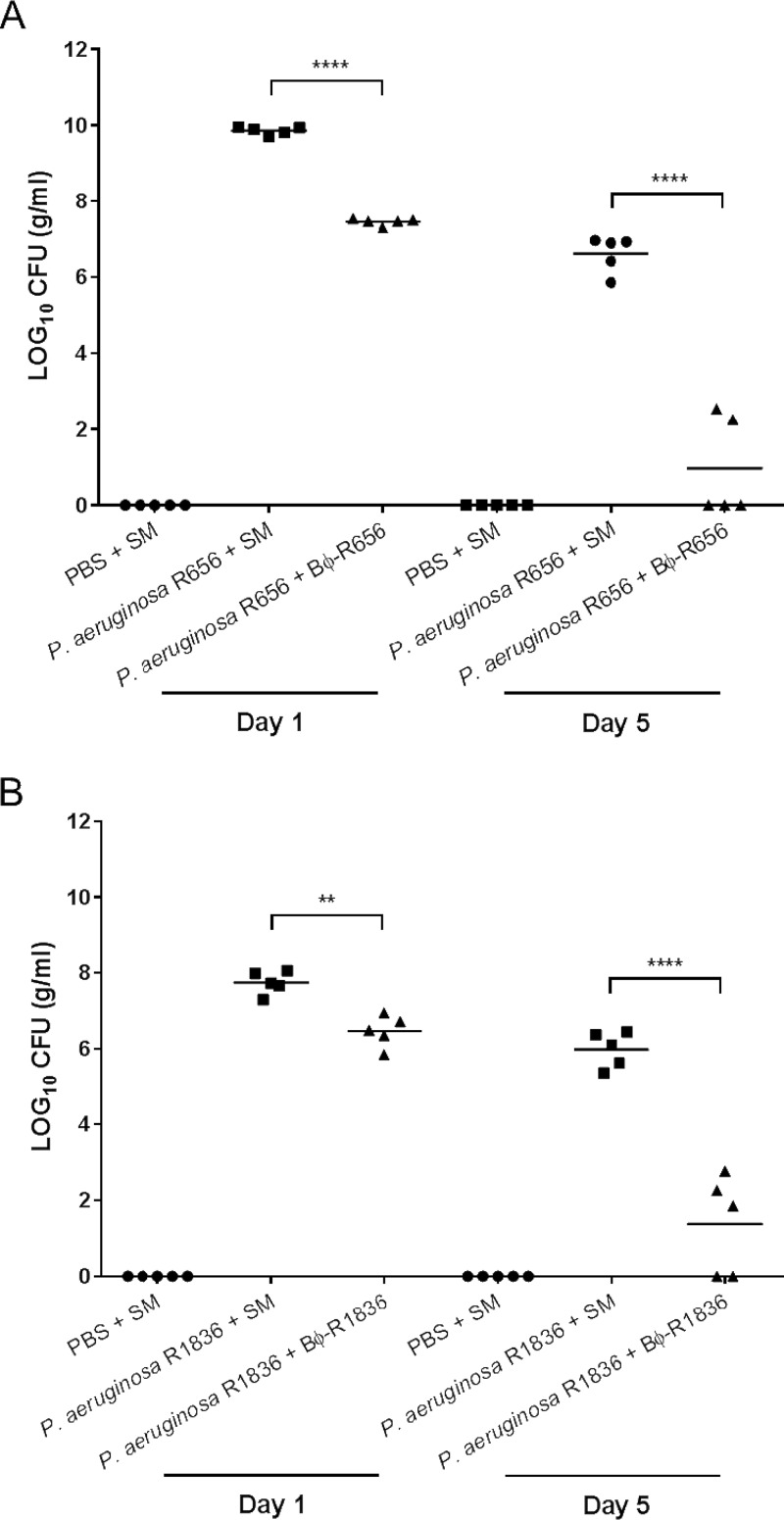
Bacterial counts in lung sections on days 1 and 5 from mice treated with Bϕ-R656 (A) or Bϕ-R1836 (B). One-way ANOVA followed by Tukey’s multiple-comparison test was used to compare the bacterial counts (****, *P* < 0.0001; **, *P* = 0.0038).

To assess the *in vivo* bacteriolytic activities of the two phages, the number of viable bacteria in the lungs of mice with acute pneumonia was measured on days 1 and 5. As shown in Fig. 7, phages Bϕ-R656 and Bϕ-R1836 exhibited excellent elimination of the XDR P. aeruginosa host strains. The bacterial load decreased by >2 log_10_ CFU (from 9.8 to 7.4 log_10_ CFU; ****, *P* < 0.0001) after treatment with phage Bϕ-R656 (Fig. 7A) and by >1 log_10_ CFU (from 7.7 to 6.4 log_10_ CFU; **, *P* = 0.0038) after treatment with phage Bϕ-R1836 (Fig. 7B) compared to the bacterium-infected group on day 1. Additionally, the phages decreased the bacterial load by >6 log_10_ CFU (from 6.6 to 0.8 log_10_ CFU; ****, *P* < 0.0001) and >4 log_10_ CFU (from 5.9 to 1.3 log_10_ CFU; ****, *P* < 0.0001), respectively, on day 5. Moreover, complete bacterial clearance was seen in some mice in the phage-treated group on day 5. No viable bacteria were detected in the lungs of the buffer- and phage-only groups (data not shown) on day 1 or 5.

## DISCUSSION

XDR Pseudomonas aeruginosa is one of the most common Gram-negative pathogens causing nosocomial infections and is a major public health concern because it possesses numerous resistance mechanisms, including beta-lactamases, carbapenemases, and efflux pumps (33, 34). As there is renewed interest in phages as therapeutic tools, we isolated two novel P. aeruginosa phages specifically targeting XDR-PA clinical isolates. The 28 XDR-PA strains used in this study were collected from various clinical samples of patients with pneumonia and exhibited resistance to most antibiotics (see Table S1 in the supplemental material). All of them were resistant to carbapenems, one of the best choices for treatments of multidrug-resistant Gram-negative bacteria, including imipenem (MIC, ≥16 μg/ml) and meropenem (MIC, ≥16 μg/ml) (Table S1). These strains showed different PFGE patterns (Fig. S1).

We initially isolated eight P. aeruginosa phages from sewage water using XDR-PA as the host bacteria. Morphology determined using TEM revealed that all phages were of the *Siphoviridae* family within the *Caudovirales* order. Two of the 8 phages, Bϕ-R656 and Bϕ-R1836, exhibited a wide host range and strong bacteriolytic activities against the P. aeruginosa clinical strains and were examined in detail *in vitro*, *in silico*, and *in vivo* to evaluate them as potential therapeutic phage agents.

In the *in vitro* experiments, Bϕ-R656 and Bϕ-R1836 exhibited a relatively broader host spectrum than other phages targeting MDR P. aeruginosa strains (35–37), and these phages showed significant growth-inhibitory activity against host XDR-PA strains. Host range is an important parameter for initial screening of new effective phages against target bacteria, and this spectrum was confirmed using both spot tests and EOP assays (38, 39).

In previous studies, the results of EOP assays have not always correlated with those of spot tests on collected pathogenic strains (38, 40). However, we found that the range and ratios of EOP values of both phages were relatively similar to the results of the plaque clarity values in the spot test against the P. aeruginosa strains used in the study (Table S1). Therefore, we suggest that these two methods may be complementarily applied to primary selection of new phages that possess broad and strong bacteriolytic activity.

In the adsorption and one-step kinetics of both phages (Bϕ-R656 and Bϕ-R1836), Bϕ-R1836 presented burst size that was approximately two times higher than that of Bϕ-R1836 (Fig. 2B) while Bϕ-R1836 adsorbed to the host cell more slowly than Bϕ-R656 (Fig. 2A). Moreover, these two phages exhibited relatively higher burst size than other P. aeruginosa phages JG024 (180 PFU per infected cell) (41) and C11 (11 PFU per infected cell) (42). Therefore, Bϕ-R656 and Bϕ-R1836 showed better potential to be used for the control of XDR-PA strains, as both of these phages could infect a large number of host bacteria at the same time, and this could also reduce the potential for the emergence of phage-resistant bacteria.

Among the physiological properties of phages, temperature and pH stability could be considered important factors for the survival of phages during infectivity and storage (43). Therefore, phages that have high stability at various temperatures and pH values could be selected as some of the best candidates for application as sanitizers or alternative therapeutic agents (44). In this study, Bϕ-R656 and Bϕ-R1836 showed higher stability than other *Pseudomonas* phages (35, 36) at various temperatures (for 1 h) and pH values (for 30 days) (Fig. S2 and S3). These data can be used for optimizing storage and therapeutic application of phages under various physiological conditions.

Biofilm formation by pathogens is a major problem, causing chronic infectious diseases that are difficult to treat in health care settings due to antibiotic resistance (45). In particular, biofilm-producing P. aeruginosa causes severe chronic lung infections in immunocompromised individuals and patients with cystic fibrosis (45, 46). Many phages also possess antibiofilm activity, and several studies on *Pseudomonas* phages that eradicate bacterial biofilms have been reported (30, 47–49). Our phages, Bϕ-R656 and Bϕ-R1836, could also efficiently remove biofilms of host XDR-PA strains (Fig. S4). The biofilm disruption ability of these phages could be used as a biocontrol agent, not only to prevent biofilm formation on medical devices in hospital environments but also to control biofilm-related infections.

The bioinformatics data of the two phage genomes revealed that phage Bϕ-R656 possesses a novel gene composition with <16% sequence similarity to other known P. aeruginosa phages in the NCBI database (Fig. 3C). On the other hand, phage Bϕ-R1836 has >91% sequence similarity to the complete genomes of known phages (Fig. 3D). However, the genomes of both phages include many unknown genes because functional identification of phage genes in the genome databases is inadequate. Nevertheless, some of the putative genes of these phages exhibited sequence relatedness and similar genomic organization to the genomes of other known phages, indicating that they evolved through horizontal exchange of genes with other phages (50, 51).

*In vivo* treatment with Bϕ-R656 and Bϕ-R1836 significantly increased the survival rate in the G. mellonella larva infection (Fig. 4) and mouse pneumonia (Fig. 5) models using XDR-PA clinical strains and reduced histologic damage to the bacterium-challenged larval tissues (Fig. 6; also Fig. S5). Recent studies have demonstrated the therapeutic efficacy of phages against P. aeruginosa strains using G. mellonella larvae (13, 52, 53). Especially, Beeton et al. (13) and Forti et al. (53) reported assessments of *in vivo* phage therapy by phage cocktails. In the application of monophages, phage KT28 at an MOI of 100 also increased the survival of G. mellonella against both non-CF and CF *pseudomonas* strains (52); however, the survival rates of phage KT28 were significantly lower than those of two phages in our study.

In our study, we found that Bϕ-R656 and Bϕ-R1836 were less effective against each other’s strains than their original host strains (Fig. 4C and D). Moreover, this result was notably similar to that of the clarity of host spectrum and EOP values of the two phages against their host strains (Table S1). These data indicate that G. mellonella larvae can be used as a surrogate model to initially screen effective phages before mouse experiments. Furthermore, because the innate immune responses of G. mellonella to pathogenic infections are very similar to those of mammals (54), G. mellonella larvae can be used as a more simplistic and manageable system than vertebrate models to evaluate infections with various pathogens (55).

To date, several studies on the therapeutic efficacy of phages against P. aeruginosa clinical strains using mouse lung infection models have been reported (26, 32, 53, 56–59). Our results also showed that treatment with the two novel phages significantly increased survival in the mouse acute pneumonia model (Fig. 5). In previous studies using monophages, treatments with PAK-P3 (56) and PAK-P1 (32) led to higher survival rates (MOI of 10 at 2 h postinfection) than our phages; however, it should be noted that the mice were treated with phage (MOI of 10) at 4 h postinfection.

In the mouse acute pneumonia model, phages Bϕ-R656 and Bϕ-R1836 significantly reduced the bacterial loads of the host bacteria in the lungs (Fig. 7). Additionally, the histologic features of each group accurately reflected the therapeutic effects of each phage in terms of survival and bacterial clearance (Fig. 6). Moreover, there were no adverse effects observed due to phage treatment in this study.

### Conclusions.

In the present study, two novel siphoviridal P. aeruginosa phages, Bϕ-R656 and Bϕ-R1836, were investigated for their *in vitro* physiological characteristics, *in silico* bioinformatic properties, and *in vivo* therapeutic effects against XDR-PA isolates from pneumonia patients using two animal models. Overall, both phages showed strong bacteriolytic activity and therapeutic efficacy *in vitro* and *in vivo* against the XDR-PA clinical strains, as well as pseudomonal biofilm disruption. This work strongly suggests that these two novel phages can be used for treating patients with pulmonary infections caused by clinical P. aeruginosa strains. Furthermore, the physiological and genetic studies of both phages provide insights to further the application of phages as therapeutic alternatives for XDR-PA infections.

## MATERIALS AND METHODS

### Bacterial strains.

The 28 XDR-PA strains were isolated from sputum, urine, and endotracheal aspirate clinical samples from pneumonia patients at a university hospital in South Korea. The XDR clinical isolates were identified using matrix-assisted laser desorption ionization–time of flight mass spectrometry (MALDI-TOF; Vitek MS system; bioMérieux, Marcy l’Etoile, France). Antimicrobial susceptibility testing of the isolates was performed using the VitekN132 system (bioMérieux) and the Clinical and Laboratory Standards Institute (CLSI) disk diffusion method (76). Pulsed-field gel electrophoresis (PFGE) with the CHEF-DR-II system (Bio-Rad Laboratories, Hercules, CA, USA) was used to determine the genomic DNA clonality of the clinical isolates, and the PFGE patterns were analyzed using InfoQuest FP software (version 4.50; Bio-Rad Laboratories, Inc.) (see Fig. S1 in the supplemental material). All collected XDR-PA clinical strains were resistant to most antibiotics tested, including the carbapenems (imipenem and meropenem). In particular, XDR-PA strains YMC11/02/R656 and YMC11/11/R1836, isolated from sputum samples of patients with pneumonia, were used as the host strains to evaluate the *in vitro* and *in vivo* therapeutic potential of phages Bϕ-R656 and Bϕ-R1836. The antimicrobial susceptibility profiles of all XDR-PA strains used in this study are listed in Table S1.

### Isolation and purification of phage infecting XDR-PA strains.

Phages capable of lysing XDR-PA clinical strains were isolated from sewage water at a hospital in South Korea as described previously (60). Briefly, a suspension of host strains, P. aeruginosa YMC11/02/R656 and YMC11/11/R1836, in 4 ml of Luria-Bertani broth (LB) medium (Difco, Detroit, MI, USA) adjusted to an OD_600_ of 0.5 was mixed with 1 ml of sewage solution and incubated at 37°C for 12 h with shaking. The lysate was centrifuged at 12,000 × *g* for 10 min at 4°C and filtered using a 0.22-μm membrane (Millipore Corporation, Bedford, MA, USA) to remove cell debris. Phage plaques were obtained using the double layer method (61). Purification of phages from single plaques was repeated at least three times using the process described above until homogeneous plaques were obtained. To concentrate the purified phages, the phage solution was treated with NaCl (final concentration, 1 M; Merck) and polyethylene glycol 8000 (final concentration, 10%; Sigma, St. Louis, MO, USA) and incubated at 4°C for 12 h. The concentrated phages were collected through ultracentrifugation at 12,000 × *g* for 1 h at 4°C and resuspended in 1 ml of sterilized sodium chloride-magnesium sulfate (SM) buffer (100 mM NaCl, 8 mM MgSO_4_, 2% gelatin, 50 mM Tris-HCl, pH 7.5). The phage stocks were titrated through plaque assays using the double layer method (61). The concentrated phage stocks in SM buffer were stored at 4°C and used for *in vitro* and *in vivo* experiments. Among the eight XDR-PA-lysing phages (Bϕ-P54, Bϕ-R24, Bϕ-R656, Bϕ-R960, Bϕ-R1561, Bϕ-R1836, Bϕ-R2928, and Bϕ-R3040) which were isolated in this study, two phages (Bϕ-R656 and Bϕ-R1836) were selected and further evaluated for their *in vitro* and *in vivo* therapeutic effects, as they exhibited wider host range and stronger bacteriolytic activity.

### Transmission electron microscopy.

The morphology of the purified phage particles was analyzed using transmission electron microscopy (TEM) as described previously (60). Briefly, concentrated phage solution was mounted on 300-mesh copper grids, stained with uranyl acetate, and analyzed using TEM (JEOL JEM-1011; Tokyo, Japan) at 80 kV.

### Adsorption and one-step growth kinetic analyses of phages.

The adsorption rate and one-step growth kinetic analyses of Bϕ-R656 and Bϕ-R1836 were performed as previously described, with some modifications (62). Briefly, for the adsorption rate test, host bacterial suspension (10^5^ CFU/ml) was mixed with phage solution at an MOI of 0.001 and incubated at 37°C. Samples of the mixture (100 μl) were taken at 1, 2, 3, 4, 5, and 10 min and centrifuged immediately (12,000 × *g*, 10 min). The titer of unadsorbed phages of supernatants was determined using the double-layer agar plate method (61). For one-step growth kinetics, host bacterial cells were cultured in 5 ml LB medium (10^5^ CFU/ml) and added to phage solutions at an MOI of 0.001. After incubation for 5 min, supernatant including free phages was removed by centrifugation (12,000 × *g*, 10 min), and pellet was resuspended in 5 ml fresh LB medium. Subsequently, culture samples (100 μl) were collected at 5-min intervals for 100 min. Phage titration of samples was immediately examined using the double-layer agar plate method (61).

### Host range of phages.

To determine the host range of isolated phages (P. aeruginosa phages Bϕ-R656 and Bϕ-R1836) against the XDR-PA clinical isolates, the spot test was used as described previously with some modifications (62). Briefly, aliquots of 5 μl phage solution (a final titer of 1 × 10^5^ PFU/ml) were serially diluted with SM buffer and spotted on lawns of various XDR-PA strains growing on Luria-Bertani (LB) agar plates, which were then incubated at 37°C for 12 h. The plaques were classified into one of three categories according to degree of clarity: clear (++), turbid (+), and no plaque (−). The efficiency of plating (EOP) for each phage on XDR-PA strains was determined using the double-layer agar plate method and was calculated as the ratio of PFU of test strain (remaining 26 XDR-PA strains) to PFU of host strain (YMC11/02/R656 and YMC11/11/R1836) (63).

### Host cell lysis and stability of phages.

Lysis assays using P. aeruginosa phages Bϕ-R656 and Bϕ-R1836 were performed as described previously (60). Briefly, the cultured host bacteria (OD_600_ = 0.2) in fresh LB medium were mixed with the phage in SM buffer at a multiplicity of infection (MOI) of 0.1, 1, or 10. The bacterial turbidity was measured at 1-h intervals for 6 h using spectrophotometry at 600 nm. The thermal and pH stabilities of the phages were determined as described previously (60), and phage titers were determined using the double-layer agar method. The thermal stability of phages was measured after incubation at 4°C, 40°C, 50°C, 60°C, or 70°C for 1 h, and pH stability was measured after 1, 2, 3, 4, or 5 days and 1 month at pH 4, 5, 6, 7, 8, 9, and 10 at 4°C. Data are presented as percentage of surviving phages, and tests were performed in triplicate.

### Biofilm formation and crystal violet assay.

Assays for biofilm formation and activity of phages against biofilms were performed as previously described, with modifications (64). For biofilm formation of host XDR-PA strains, 200 μl of YMC11/02/R656 or YMC11/11/R1836 bacterial suspension was diluted to an OD_600_ of 0.2 in LB broth, dispensed into each well of a sterile 96-well flat-bottomed polystyrene plate (Corning, New York, USA), and cultured for 48 h at 37°C. Negative-control wells were filled with LB broth only. Plates were sealed to avoid water loss by evaporation, and medium was renewed every 12 h. After incubation for 48 h, plates were gently washed three times with PBS to remove cell debris, and 200 μl of LB broth, either alone or containing phage at 1 × 10^7^ PFU/ml, was applied to each well and incubated at 37°C for 12 h. Supernatant was then removed, and each well was gently washed three times with PBS and air dried for 1 h. Plates were stained with 200 μl of 0.1% crystal violet solution (Merck) in PBS for 10 min, washed with PBS, and air dried for 1 h. Crystal violet dye was then eluted with 125 μl of 30% (vol/vol) glacial acetic acid for 10 min. The absorbance of each well was measured at 590 nm using UV spectrophotometry, and each experiment was repeated three times.

### Phage whole-genome sequencing and bioinformatics analysis.

The phage DNA was extracted and purified according to the standard phenol-chloroform extraction protocol as described previously (65). Whole-genome sequencing was conducted at ChunLab, Inc. (Seoul, South Korea), using a 454 GS Junior genome analyzer (Roche Life Sciences, Branford, CT, USA), and *de novo* assembly was carried out using Roche gsAssembler (version 2.6; Roche) and CLC Genomics Workbench (version 4.8; CLC bio, Aarhus, Denmark). Open reading frames (ORFs) were predicted using the NCBI ORF finder (66) and GenMark.hmm software (67). The putative functions of the predicted ORFs were identified using BLASTP (E values of <0.1) (68) and PSI-BLAST (E value of <0.005) (http://www.ebi.ac.uk/Tools/sss/fasta/) from the NCBI database (https://www.ncbi.nlm.nih.gov/). Genomic organizations and comparisons of phages were generated using DNAPlotter (69) and Easyfig software (version 2.1) (70), respectively. The tRNA genes were predicted using the tRNAscan-SE program (71).

### Phage therapy assay in a G. mellonella infection model.

To evaluate the therapeutic efficacy of Bϕ-R656 and Bϕ-R1836 against XDR-PA strains, a Galleria mellonella larva infection was used as described previously, with some modifications (72). G. mellonella larvae weighing 200 to 250 mg were selected randomly and swabbed with 70% ethanol. They were fasted in a 90-mm petri dish in darkness at 37°C for 24 h prior to the test. The larvae were divided into the following 6 groups of 10 larvae each: (i) control, PBS (phosphate-buffered saline; Invitrogen) + SM; (ii) bacterial infection, 1 × 10^5^ CFU/ml; (iii) phage-only, 1 × 10^10^ PFU/ml; (iv), phage treatment at an MOI of 100, 1 × 10^7^ PFU/ml; (v) phage treatment at an MOI of 10, 1 × 10^6^ PFU/ml; and (vi) phage treatment at an MOI of 1, 1 × 10^5^ PFU/ml. A bacterial amount of 5 μl (5 × 10^2^ CFU) was injected into the last right-side proleg of the larvae using a 10-μl Hamilton syringe (701RN; Hamilton Bonaduz AG, Bonaduz, Switzerland), and the phages at 5 μl (5 × 10^4^ PFU) were injected into the last left-side proleg at 1 h postinfection. The larvae were then incubated in the dark at 37°C in 90-mm plastic petri dishes. To compare the therapeutic efficacies between Bϕ-R656 and Bϕ-R1836, larvae injected with host bacteria at the same concentration were treated with different phages, and the survival of the larvae was determined every 8 h for 72 h. The larvae were considered dead when there was no movement in response to touch. All experiments were repeated three times.

Histological analysis of larvae was performed as previously described, with modifications (73). Briefly, G. mellonella larvae at 72 h were fixed in 10% formalin for 5 days and embedded in paraffin. Tissues were stained with hematoxylin and eosin (H&E), and morphological features of tissues were observed using an optical microscope.

### Phage therapy assay in a mouse acute pneumonia model.

The efficacy of phage therapy in a mouse acute pneumonia model caused by XDR-PA clinical strains was determined as previously described (74). Briefly, C57BL/6 mice (female, aged 7 to 8 weeks) were randomly divided into the following six groups (*n* = 5 per group): buffer-only inoculation (PBS + SM) group, bacterium-only infection (1 × 10^8^ CFU/ml) group, phage-only treatment (1 × 10^9^ PFU/ml) group, and phage treatment (1 × 10^9^ to 1 × 10^7^ PFU/ml for MOIs of 10, 1, and 0.1). All mice were intraperitoneally injected with cyclophosphamide (200 mg/kg of body weight; Sigma-Aldrich) at 48-h intervals before infection (75). Mice were intranasally treated with 30 μl of phage (MOI = 10, 1 and 0.1) or SM buffer at 4 h after either administration of 30 μl of bacteria (1 × 10^8^ CFU/ml) or PBS via the same route. All mice were anesthetized via intraperitoneal injection of Zoletil-Rompun before each intranasal procedure. The mortality and body weights of the mice were recorded for 12 days.

For bacterial counts in the lung, histology, and cytokine analyses, non-cyclophosphamide-treated mice were randomly divided into the following four groups (*n* = 10 per group): buffer-only inoculation (PBS + SM) group, bacterium-only infection (1 × 10^8^ CFU/ml) group, phage-only treatment (1 × 10^9^ PFU/ml) group, and phage treatment (1 × 10^9^ PFU/ml for MOI = 10). None of the groups was injected with cyclophosphamide, and their body weights were measured for 12 days. After bacterial infection or phage treatment, five mice from each group were sacrificed on day 1 and the remaining five on day 5. To determine the number of viable bacteria in the lungs, homogenates of the collected lungs were serially diluted in PBS and plated on LB agar plates containing ampicillin (50 μg/ml). Lung histology assays were performed as previously described with modifications (56). Briefly, the right lung tissues were fixed in 10% formalin for 48 h and embedded in paraffin. The 3-μm-thick tissue sections were stained with H&E and analyzed using an optical microscope. To evaluate immunogenicity against the bacteria and phages, the levels of tumor necrosis factor alpha (TNF-α) and interleukin 6 (IL-6) in the lung lysates were quantified with the commercial DuoSet kit (R&D Systems).

### Ethics statement.

All animal experiments followed the regulations of the Institutional Animal Care and Use Committee of Yonsei University College of Medicine, Seoul, South Korea (IACUC approval no. 2014-0031-2).

### Statistical analysis.

Statistical analyses were performed with GraphPad Prism software (version 6; San Diego, CA, USA). The log rank (Mantel-Cox) test was used for survival curves. One-way ANOVA followed by Tukey’s test was used to compare bacterial counts and cytokine levels. Differences in biofilms were analyzed using the two-tailed *t* test, and *P* values of <0.05 were considered significant.

### Accession number(s).

The complete genomes of Bϕ-R656 and Bϕ-R1836 were deposited into the GenBank database under accession numbers KT968831.1 and KT968832.1, respectively.

## Supplementary Material

Supplemental file 1

Supplemental file 2
