# Supplementary material for: Two Novel Bacteriophages Improve Survival in Galleria mellonella Infection and Mouse Acute Pneumonia Models Infected with Extensively Drug-Resistant Pseudomonas aeruginosa
Source: Appl Environ Microbiol. 2019 Apr 18;85(9):e02900-18. doi: 10.1128/AEM.02900-18 (PMC6495756; doi:10.1128/AEM.02900-18)
Supplement: Supplemental file 1 [file AEM.02900-18-s0001.pdf]

Two Novel Bacteriophages Improve Survival in *Galleria mellonella* Infection and Mouse Acute Pneumonia Models Infected with Extensively-drug-resistant *Pseudomonas aeruginosa*

Supplementary Method

Pulsed-field gel electrophoresis (PFGE) analysis

PFGE analysis was performed as described previously (1). Briefly, *Xba*I-digested genomic DNAs of XDR-PA clinical strains were prepared according to the instruction of Bio-Rad (Hercules, Calif.). DNA fragments were placed on a 1 % agarose gel and separated for 20 h at 6 V/cm using a CHEF-DR II system (Bio-Rad, Hercules, CA). The dendrogram of the PFGE band patterns of the *P. aeruginosa* isolates was analyzed using InfoQuest FP software (version 4.50, Bio-Rad Laboratories, Inc) (Figure S1).

References

1. **Lee K, Lim JB, Yum JH, Yong D, Chong Y, Kim JM, Livermore DM.** 2002. blaVIM-2 Cassette-Containing Novel Integrons in Metallo-beta-Lactamase-Producing *Pseudomonas aeruginosa* and *Pseudomonas putida* Isolates Disseminated in a Korean Hospital. Antimicrob Agents Chemother **46:**1053-1058. http://dx.doi.org/10.1128/aac.46.4.1053-1058.2002.
